# Reuse of Pillaring Agent in Sequential Bentonite Pillaring Processes

**DOI:** 10.3390/ma10070705

**Published:** 2017-06-27

**Authors:** Francine Bertella, Sibele B. C. Pergher

**Affiliations:** Laboratory of Molecular Sieves—LABPEMOL, Institute of Chemistry, Federal University of Rio Grande do Norte—UFRN. Av. Senador Salgado Filho, 3000, Lagoa Nova, University Campus, 59072-970 Natal-RN, Brazil; francinebertella@gmail.com

**Keywords:** pillaring solution, Keggin ion, reutilization

## Abstract

This work describes the synthesis and characterization of pillared clays using a new pillaring method: the reuse of the pillaring solution. First, an Al pillared clay (PILC) was synthesized, and after filtration, the pillaring agent was stored and reused for an additional three pillaring procedures (P1, P2, and P3). The filtered pillaring solution was stored for one year and then reused for one additional pillaring procedure (P4). The samples were analyzed using XRD, N_2_ physisorption measurements and chemical analysis (EDX). All of the samples exhibited basal spacings larger than 17 Å and BET surface areas greater than 160 m^2^/g. After the P4 pillaring, the pillaring agent was precipitated with a Na_2_SO_4_ solution, and the resulting solid was analyzed using XRD and SEM. The results indicated that even after a total of five pillaring procedures, Al_13_ ions were still present in solution. Therefore, it is possible to reuse the pillaring solution four times and to even store the solution for one year, which is important from an industrial perspective.

## 1. Introduction

Pillaring is the process by which a layered compound is transformed into a thermally stable micro- and/or mesoporous material with retention of the layered structure. The obtained material is a pillared compound or a pillared layered solid. Pillared clays (PILCs) constitute a special class of pillared layered solids. The goal of the pillaring process is to introduce micro- and mesoporosity into clay minerals. This is achieved using a combination of a smectite, for example, and a pillaring agent via an ion-exchange reaction, in which a two-dimensional channel network is formed [1]. Heating the clays imparts stability to the pillared clay by promoting permanent bonding between the pillar and the layers. The resulting materials have small cavities and a large surface area. These properties, along with their low cost, make pillared clays ideal for use as alternative catalysts to zeolites [2].

There are numerous oligomeric cations that could be used as pillars, as well as Al, Zr, Ti, Cr, Fe, Ce and others [1,3,4]. These cations could be used individually [2,5,6,7,8] or mixed [9,10,11,12,13,14,15,16,17]. In addition, there is an enormous amount of work dealing with the use of Al-pillared clays as supports of metal nanoparticles (Pd and Pt, for example) for several catalytic applications [18,19,20,21].

In this sense, most of the studies published to date have focused on the use of the Al polyoxocation as a pillaring agent. Solutions containing this complex are prepared through forced hydrolysis, either by the addition of a base to AlCl_3_ or Al(NO_3_)_3_ solutions, up to an OH/Al molar ratio of 2.5, or by dissolution of Al powder in AlCl_3_ [22]. The most important cationic complex formed is most likely the polynuclear [Al_13_O_4_(OH)_24_(H_2_O)_12_]^7+^, which is the so-called Keggin or Al_13_ ion [23,24]. Although the method of preparation is clearly of prime importance, many other factors influence the formation of pure Al_13_ in solution, such as the nature and initial concentration of the reagents used, the degree of hydrolysis (OH/Al ratio), the rate of adding the reactants, the temperature, and the time and temperature of aging the hydrolyzed solutions [25].

Several methods and pillaring solutions, species of pillars and types of clays have been discussed in the literature. Nevertheless, most of these studies were performed using pillared clays prepared at the laboratory scale, usually in small amounts. However, some authors have performed experiments using concentrated clay dispersions [13,26,27,28,29,30,31,32]. All of these studies used Al_13_ as a pillaring agent or Al_13_ mixed with another cation, such as Fe. However, to our knowledge, none of the works reported in the literature reused the pillaring agent more than once. Thus, the purpose of this investigation is to reuse the pillaring agent in other pillaring procedures for scale-up studies.

## 2. Materials and Methods

### 2.1. Synthesis of the Aluminum Pillared Clay Minerals

The clay used in this study is a bentonite that contains montmorillonite as the dominant clay mineral. The first step of the pillaring process is to prepare the pillaring agent. For this purpose, 500 mL of a 0.2 mol/L sodium hydroxide solution (NaOH, Vetec Fine Chemicals, Duque de Caxias, Brazil) and 250 mL of a 0.2 mol/L aluminum chloride hexahydrate solution (AlCl_3_·6H_2_O, Fluka Analytical, Sigma-Aldrich, St. Louis, MO, USA) were used. The sodium hydroxide solution was slowly added to the aluminum chloride solution dropwise, which was then maintained at room temperature under stirring for six days. A ratio of 0.05 moles of Al/g of dry clay and a molar ratio of OH/Al = 2 [33] were used. 

The clay dispersion was prepared by adding 3 g of clay to 300 mL of distilled water, followed by stirring at room temperature for 2 h. After this expansion process step, the pillaring process itself was performed by adding the pillaring agent solution to the clay dispersion. The mixture was stirred for 2 h at room temperature such that the natural cations of the clay could be exchanged for the previously prepared oligomers, thereby affording an intercalated clay. The solid was separated by vacuum filtration, washed with distilled water until the test for chloride was negative, dried at 60 °C and subsequently calcined at 450 °C to generate the pillared clay. 

During the filtration step, the pillaring agent was separated. The separated pillaring agent was stored in an amber bottle and subsequently reused in three other pillarings—called P1, P2 and P3—as shown in Table 1. After filtration of the P3 clay, the pillaring agent was again stored in an amber bottle, and it was stored for one year. After this period, the pillaring agent was reused for pillaring P4. All other pillaring steps were performed as described above. 

### 2.2. Preparation of A1_13_ Sulfate

The methodology used was based on [13] and [23] and is described below. After pillaring P4, the pillaring agent filtrate was added to a Na_2_SO_4_ (Vetec Fine Chemicals, Duque de Caxias, Brazil) solution (500 mL of 0.1 mol/L) to precipitate the Keggin ions. The mixture was initially stirred for 2 h, followed by aging for 48 h. Then, the solid was vacuum filtered, washed with distilled water, and dried in an oven at 60 °C.

### 2.3. Material Characterization

The natural and pillared clays were characterized using XRD, textural analysis by N_2_ adsorption, chemical analysis and scanning electron microscopy. X-ray diffraction analyses were performed on a Rigaku Desktop MiniFlex II apparatus (Rigaku, Tokyo, Japan) using CuKα radiation (λ = 1.54 Å). The analyses were performed over the range of 1.5° to 65° 2θ using an X-ray tube voltage of 30.0 kV and current of 15.0 mA, scan rate of 5°/min and step size of 0.05. The textural analyses of the samples by N_2_ adsorption were conducted using the adsorption/desorption of nitrogen with a Nova 2200 E device (Quantachrome, Boynton Beach, FL, USA). The samples were pre-treated for 3 h at 300 °C under vacuum prior to the analyses. Then, the adsorption/desorption of N_2_ at liquid nitrogen temperature was measured. Chemical analyses of the samples were performed using an EDX-720 energy dispersive X-ray fluorescence spectrometer (Shimadzu, Kyoto, Japan). Characterization by scanning electron microscopy was performed using an ESEM-XL30 instrument (Philips, Eindhoven, The Netherlands). The samples were coated with gold to avoid the appearance of charges on the surface that could lead to distortions in the images.

## 3. Results and Discussion

**Figure 1:**
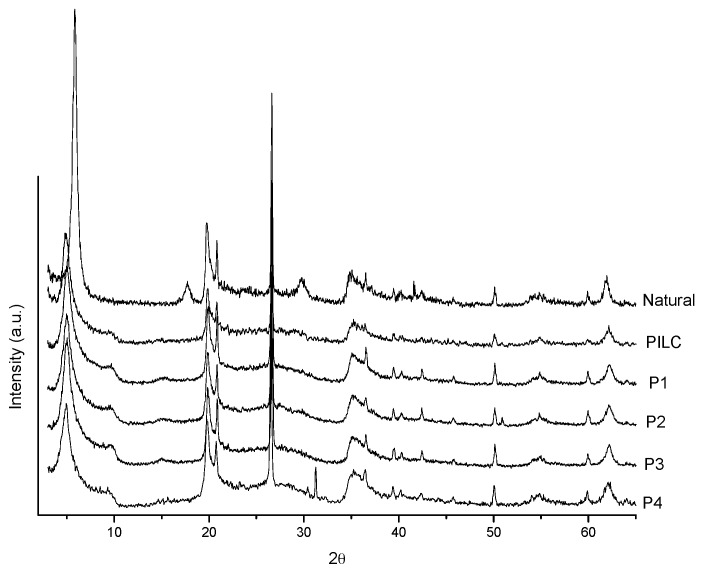
X-ray diffraction patterns of natural and pillared clays.

Figure 1 presents the X-ray diffraction patterns of the starting clay (natural) and of the pillared clays. The PILC sample refers to the first pillaring, and P1, P2, P3, and P4 refer to samples obtained from reuse of the pillaring agent. It was observed that all samples were pillared due to the increase in the interlayer spacing relative to that of the natural clay (Table 2). The starting clay had a basal spacing of 15.12 Å, which is an indication that Ca^2+^ ions are present as interlayer cations [34]. For the pillared samples, basal spacings of approximately 17.8 Å were obtained. This basal spacing is in agreement with those reported in the literature for pillaring with Al [5,13,35,36]. This value refers to the sum of the thickness of the lamella with the approximate size of polyoxocation [Al_13_]^7+^ (9 Å) [25], which confirms the inclusion of Al pillars between the clay lamellae.

As shown in Figure 1, a shoulder also appeared on the peak at 9° 2θ, which corresponds to a basal spacing of approximately 9.6 Å. This spacing is characteristic of calcined clay, in which the layers are collapsed. This method of pillaring produces a pillared material, but some lamella may be collapsed.

The pillaring agent used in pillarings P1 to P4 was always the same; however, it was more dilute with each reuse. Nevertheless, the basal spacings of these samples were similar to that of the first pillared clay obtained using this pillaring agent (PILC). This result indicates that there are still Keggin ions (Al_13_) present in the remaining solution, which may be due to two reasons: (1) at each pillaring, 100% of the cation-exchange capacity (CEC) of the clay is not being achieved and (2) the amount of Keggin ions present in the pillaring agent is considerably greater than the exchange capacity of the clay.

Using the following equation, we can calculate the mass of Keggin ions (theoretical) required to obtain 100% cation exchange using 3 g for the mass of clay used and 1.039 g/mmol for the molar mass (theoretical) of the Keggin ion [AlO_4_Al_12_ (OH)_24_∙(H_2_O)_12_]^7+^. The cation-exchange capacity of this clay is 1.79 mmol/g [37], but we have to take into account the stoichiometry of the reaction: Keggin ions have a charge +7 and Na ions have charge +1. Because of this, 0.255 mmol/g is the CEC correct value in this case. 

Mass = mass_clay_ × CEC × factor × molar mass

Mass = 3 g × 0.255 mmol/g × 1 (100% CEC) × 1.039 g/mmol

Mass = 0.79 g

In theory, this mass is equivalent to 0.000765 moles of Keggin ions. For the synthesis of the pillaring agent, 0.05 moles of Al/g of clay are used. Because the majority of Al species present in solution are Keggin ions for the synthesis conditions for the pillaring agent used in this study [38,39], one can consider that, when using 0.05 moles, much of this amount is related to the number of moles of Keggin ions present in the pillaring agent. In fact, when using the above equation with a factor of 5 (in the five pillaring procedures that this pillaring agent was used, 100% cation exchange occurred), the number of moles of Keggin ions that would be required is 0.0038. Therefore, this amount of 0.05 moles of Al/g of clay was sufficient to perform five pillarings with 3 g of clay each.

Table 3 presents the data from the chemical analyses of the pillared clays and the starting clay. An increase in the Al content of the pillared samples relative to natural clay was observed, indicating that Al oligomers are being incorporated into the interlayer space of the clay. Notably, the Al content remained high even after one year (P4). Regarding the Si/Al ratio, a decrease in this ratio for pillared clays occurs in comparison to the natural clay, which confirms that the pillaring process occurred. It is also observed that the ratios obtained for samples P1 to P4 were similar, indicating that the pillaring agent can be reused four times. The Ca content in the pillared clays decreased relative to that of the natural clay, and in most samples, this element could not be detected. This was due to the cation exchange of Ca by oligomers. The Si content in the pillared clays was less than that in the starting clay. This difference is due to the aluminum inserted in the samples by changing the relative percentage. Regarding the values for Mg, the pillared clays exhibited a decrease compared to the natural clay, which indicates that part of the Mg ions also participate in the process and should be located in the interlayer space. The Fe content in the pillared clays increased relative to that in the natural clay, which may be associated with the technique because the EDX analysis is punctual.

**Figure 2:**
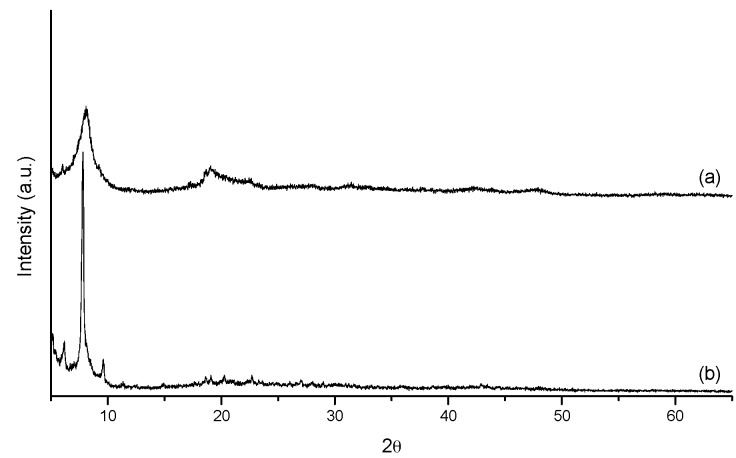
X-ray diffraction patterns of Keggin ion synthesized before being used as a pillaring agent (**a**) and after five pillaring procedures (**b**).

To verify that the pillaring species was actually the Keggin ion, the pillaring solution of the P4 sample was filtered and then added to a solution of Na_2_SO_4_ to precipitate the remaining Keggin ions. Figure 2 shows the XRD analyses of the solid precipitate (b) and of the Keggin ion synthesized using the same method before being used as a pillaring agent (a).

**Figure 3:**
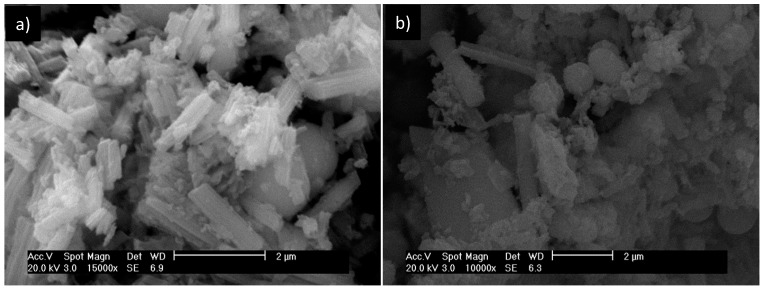
SEM micrographs of Keggin ion precipitates (**a**) before pillaring and (**b**) after five pillaring procedures.

As shown in Figure 2, both samples exhibit the same diffraction peaks but with different intensities. Similar results were obtained by Tsuchida et al. [40], in which a solid precipitate from solutions of Al_13_ was obtained by adding sulfate salts with two types of crystals (I and II). The results of this study are similar to those of the type II crystals. Tsuchida et al. [40] also found that Keggin ion solutions synthesized using OH/Al ratios equal to 2 (the basicity used in this study) preferentially generate the type II crystals when precipitated with sulfate. Thus, it appears that even after five pillaring procedures, there are Keggin ions present in the pillaring agent. The method of precipitating Keggin ions with sulfate anions exerts a strong influence on the morphology of the solid Al_13_ [41]. Figure 3 presents scanning electron microscopy images of the precipitated Keggin ions before being used (a) and after five pillaring procedures (b).

Before pillaring (Figure 3a), the precipitated Keggin ions present a morphology similar to a fiberboard cylinder. After pillaring (Figure 3b), the precipitated Keggin ions exhibit different morphologies, with some particles possessing cylindrical shapes, others with spherical shapes and even amorphous agglomerates. Wang and Muhammed [41] found different morphologies (in the form of fibers, rectangles, and tetrahedrons) when precipitating the sulfate Keggin ion by changing only the precipitation time, and the tetrahedral morphology (as reported by [23]) was only obtained after long periods of precipitation, which demonstrates that the method employed to precipitate the Keggin ions exerts a great influence on the morphologies of the crystals. 

**Figure 4:**
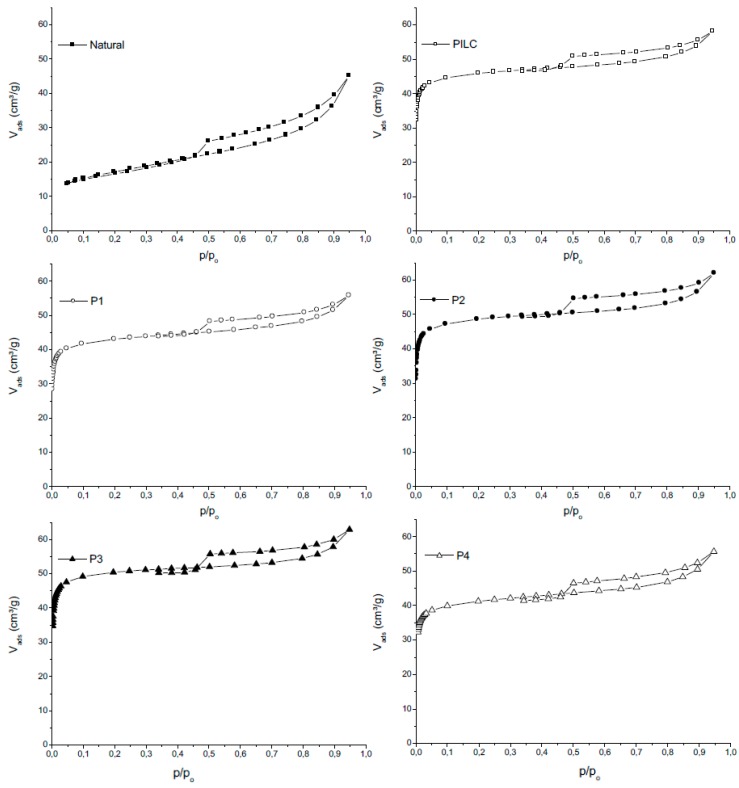
N_2_ adsorption/desorption isotherms for natural clays, PILC, P1, P2, P3, and P4.

The N_2_ adsorption/desorption isotherms are shown in Figure 4. It was observed that all samples belong to the type IIb classification according to Rouquerol et al. [42], which is characteristic of materials that contain aggregates of plate-shaped particles, typical of clays. Regarding the type of hysteresis, all can be classified as type H3, which is characteristic of porous materials that consist of agglomerates of particles in the form of plates (lamellae) that produce slit-shaped pores [43].

In comparing the isotherms of the pillared clays with the isotherm of the natural clay, it is observed that the treatment of natural clay with the Al oligomeric solution causes a considerable increase in the amount of adsorbed N_2_. For *p*/*p*_0_ = 0.2, the amount of N_2_ adsorbed by the natural clay is 17.1 cm^3^/g, whereas for pillared clay P2 (the second time that the pillaring agent was reused), for example, the amount of N_2_ adsorbed is 49 cm^3^/g, i.e., an approximately three-fold increase.

The isotherm data can be used to calculate the specific surface areas and pore volumes of the samples. The specific surface area was calculated using the BET method, and the total volume was calculated at a partial pressure of *p*/*p*_0_ = 0.95. The area and volume of micropores were calculated using the t-plot method. The external surface is the difference between the BET surface area and micropore area, and it explains the contribution of meso- and macropores. These data are presented in Table 4.

A significant increase in the BET specific surface areas of the samples is observed after the pillaring, in which the BET areas of the pillared clays are approximately four times greater than the BET area of natural clay. Furthermore, note that the largest contribution to the high values of specific area are related to the increase in the area of micropores in the material, which is characteristic of pillared clays [33,44]. The total pore volume also increases following the pillaring procedure. With respect to the microporosity, there is an approximately 10-fold increase in microporosity in the pillared clays relative to that of the natural clay. Due to this increase in micropore volume, the micropore area of these materials is approximately 10 times greater than that of the natural clay.

The analysis of the results indicated that the specific surface areas of the samples that were pillared by reusing the pillaring agent from the PILC sample remained high, which demonstrates that the pillaring solution can be reused up to four times and can also be stored for long periods of time (P4).

## 4. Conclusions

The pillaring agent can be reused in other pillaring procedures without a loss in the primary characteristics of pillared clays, i.e., both the pillared clay obtained using the traditional method (PILC) and the pillared clays obtained by reusing the pillaring agent exhibited an increase in their basal spacings and high specific areas. Even the P4 sample, in which the pillaring agent was stored for one year before being reused for the fourth time, presented results that were as good as those for the clay pillared using the traditional method. Additionally, the XRD and SEM analyses revealed that there are Al_13_ ions present in solution, even after five pillarings, which makes the reuse of the pillaring agent possible.

## Figures and Tables

**Table 1:** Data on pillaring with reuse of the pillaring agent.

Sample	Reuse of Pillaring Agent
PILC	---
P1	First time
P2	Second time
P3	Third time
P4	Fourth time (after one year)

**Table 2:** Basal spacings obtained for the pillared clays and natural clay.

Samples	d_001_ (Å)
Natural	15.12
PILC	17.86
P1	17.86
P2	17.83
P3	17.54
P4	17.90

**Table 3:** Chemical analyses of samples (%).

Sample	SiO_2_	Al_2_O_3_	MgO	CaO_2_	Fe_2_O_3_	Si/Al
Natural	69.96	19.08	5.61	2.73	2.62	3.52
PILC	59.37	33.87	3.59	n.d. ^a^	3.17	1.69
P1	60.28	30.45	3.38	n.d.	5.89	1.90
P2	60.08	30.56	3.37	0.03	5.96	1.89
P3	60.15	30.53	3.18	n.d.	6.14	1.90
P4	59.86	30.76	3.36	n.d.	6.02	1.87

^a^ n.d. = not detected.

**Table 4:** Textural parameters calculated from the adsorption isotherms for the prepared samples.

Sample	S_BET_ (m^2^/g)	S_micro_ (m^2^/g)	S_ext_ (m^2^/g)	V_micro_ (cm^3^/g)	V_total_ (cm^3^/g)
Natural	58	19	39	0.010	0.070
PILC	183	171	12	0.066	0.090
P1	171	158	13	0,062	0.086
P2	194	183	11	0.071	0.096
P3	202	192	10	0.074	0.097
P4	164	150	14	0.058	0.086
